# Technical Difficulties of Removing Huge Bilateral Breast Fibroadenomas

**DOI:** 10.7759/cureus.71822

**Published:** 2024-10-18

**Authors:** Mohamed A Ali, Mahmoud Mosbah, Hasnaa Mesbah

**Affiliations:** 1 Surgical Oncology, Cairo University/National Cancer Institute, Cairo, EGY; 2 General Medicine, Cairo University/National Cancer Institute, Cairo, EGY

**Keywords:** breast cancer, breast surgery, giant fibroadenoma, oncology, plastic and reconstructive surgery

## Abstract

We are reporting here a case of huge bilateral fibroadenomas in a young nulliparous woman with greatly enlarged breasts; the difficulty was how to remove ten huge fibroadenomas from the left breast and eight fibro adenomas from the right, in addition, the left breast was larger and more ptotic than the right. We decided to excise all the fibro adenomas through bilateral round block mammoplasty, aiming for preservation of breast tissue, normal lactation, and the desired cosmetic results instead of a bilateral subcutaneous mastectomy with implant reconstruction.

## Introduction

Fibroadenomas are the most common benign swelling of the breast in adolescent women. In young women, the overall incidence of fibroadenoma is 2.2% [1]. Most cases present as a unilateral single rubbery and well-defined breast lump. Multiple breast fibroadenomas are not common and are usually seen in 15% to 20% of women as less than four masses, usually in a single breast [2].

## Case presentation

The patient was a nulliparous woman in her second decade who presented with bilateral multiple breast swelling for two years. Her menarche was at the age of 12 years, and she had a regular cycle; there was no history of oral contraceptive pills use or family history of breast cancer. She had no other comorbidities. Clinical breast examination revealed multiple bilateral discrete masses that were well-circumscribed, freely mobile, and largest on the left side at 12 x 10 cm. The left breast was more pendulous and larger than the right. Her BMI was less than 22 kg/m^2^, and her bra size was 40 A.

**Figure 1:**
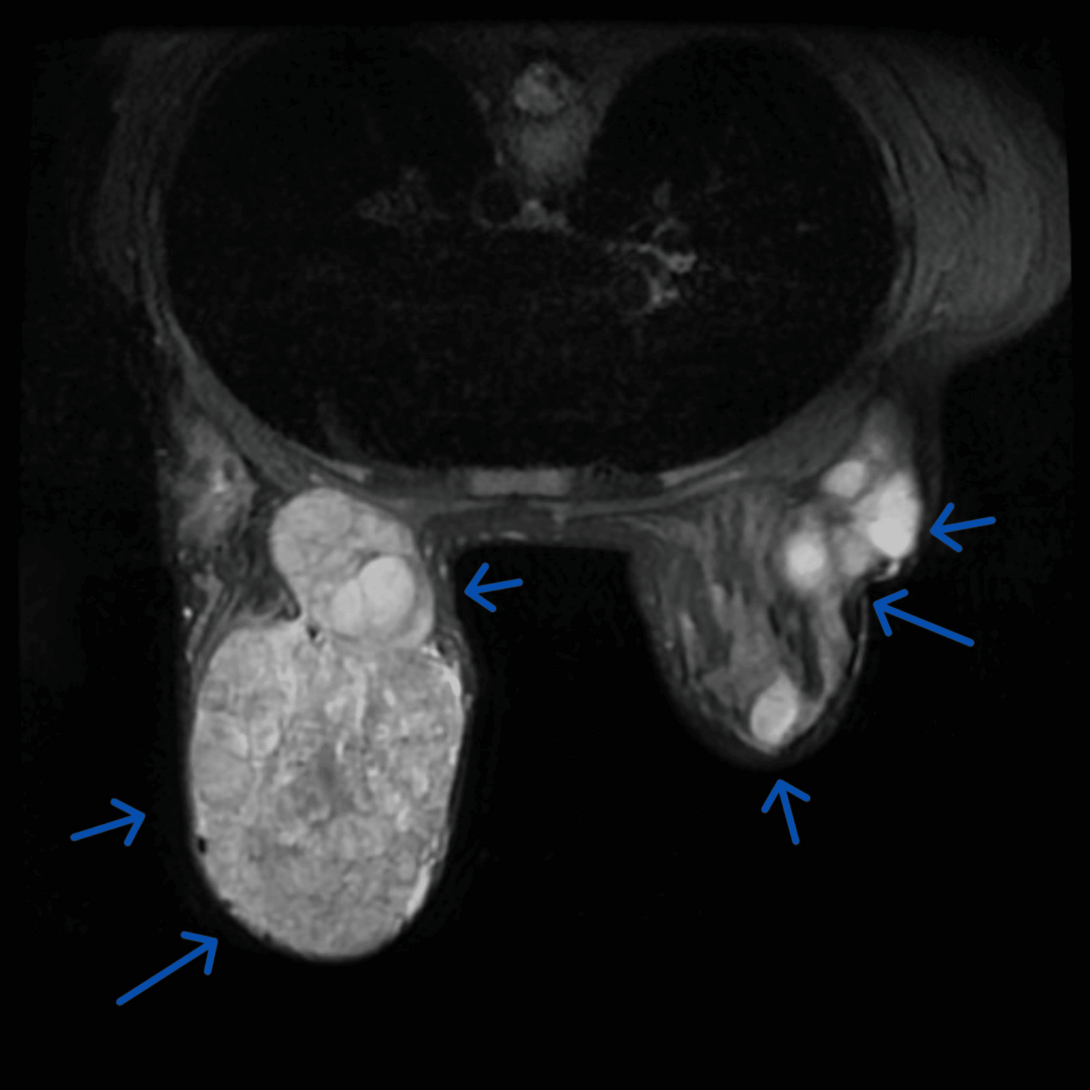
MRI image of both breasts The image shows multiple fibroadenomas occupying the whole breast tissue. Arrows show bilateral huge breast masses occupying and compressing the rest of the breast tissue.

A breast ultrasound scan revealed multiple well-defined echogenic masses with lobulation at different positions, which was in keeping with multiple fibroadenomas. MRI revealed bilateral masses almost replacing the whole breast volumes. The largest mass almost replaced the left breast. It measured 11 X 11 X 8 cm and showed central non-enhancing areas denoting necrosis (Figure 1). Ultrasound-guided true-cut biopsies from the masses indicated fibroadenomas.

**Figure 2:**
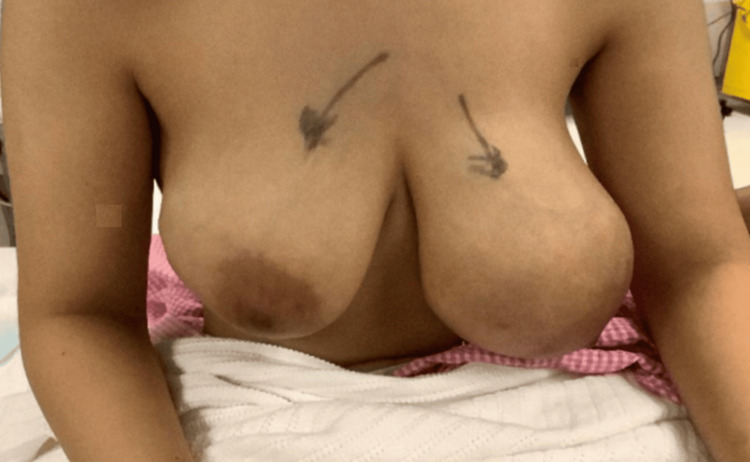
Preoperative image

Currently, the treatment of fibro adenoma is conservative or surgical excision; however, the challenges in this case were the surgical approach, preservation of normal breast tissue, restoration of the normal shape of both breasts, and excision of the excessive redundant skin (Figure 2).

**Figure 3:**
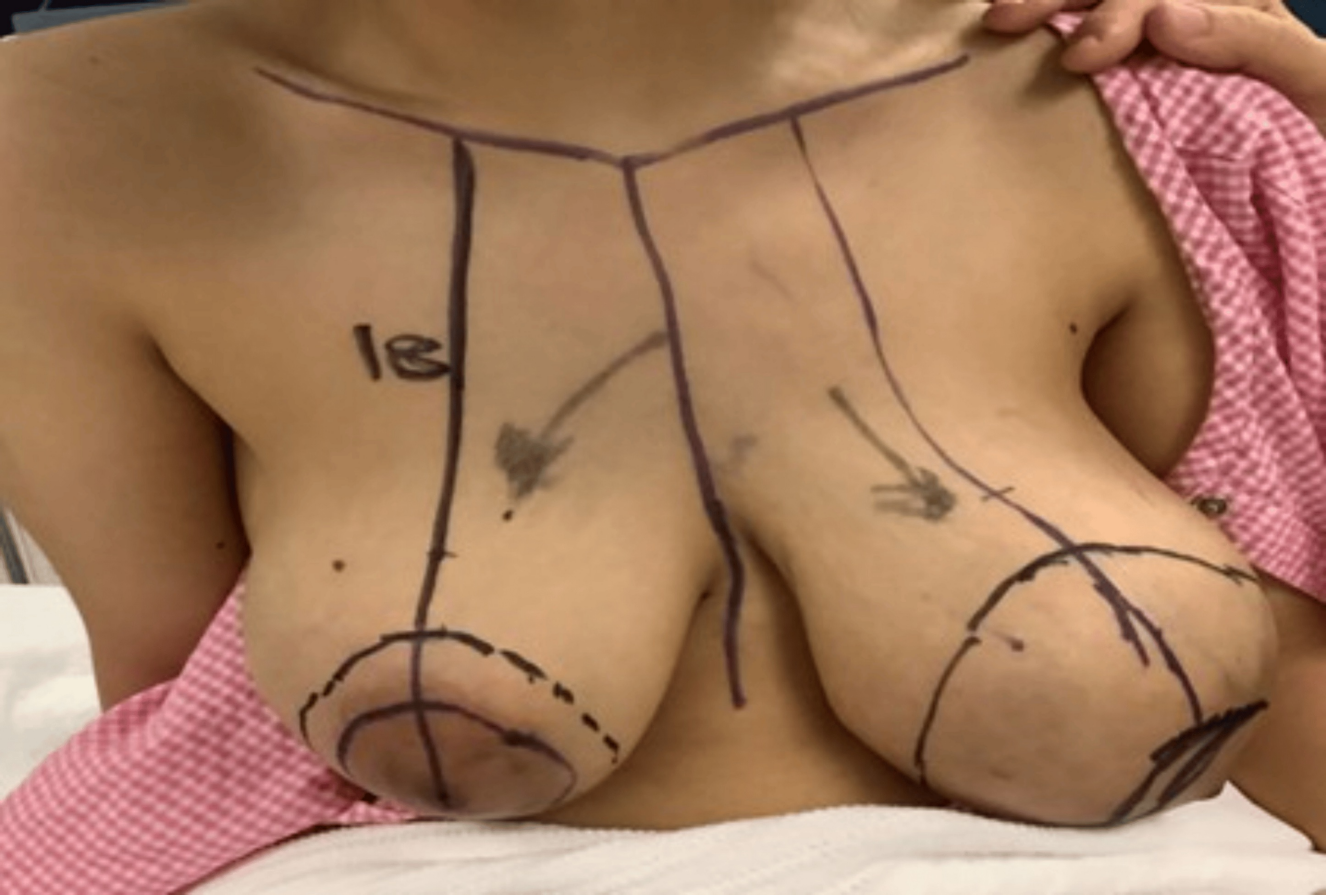
Drawing and planning for incision

We excised these masses through a bilateral round block mammoplasty under general anesthesia to conserve the breast tissue. Preoperative skin incision marking was done while the patient was standing (Figure 3). 

**Figure 4:**
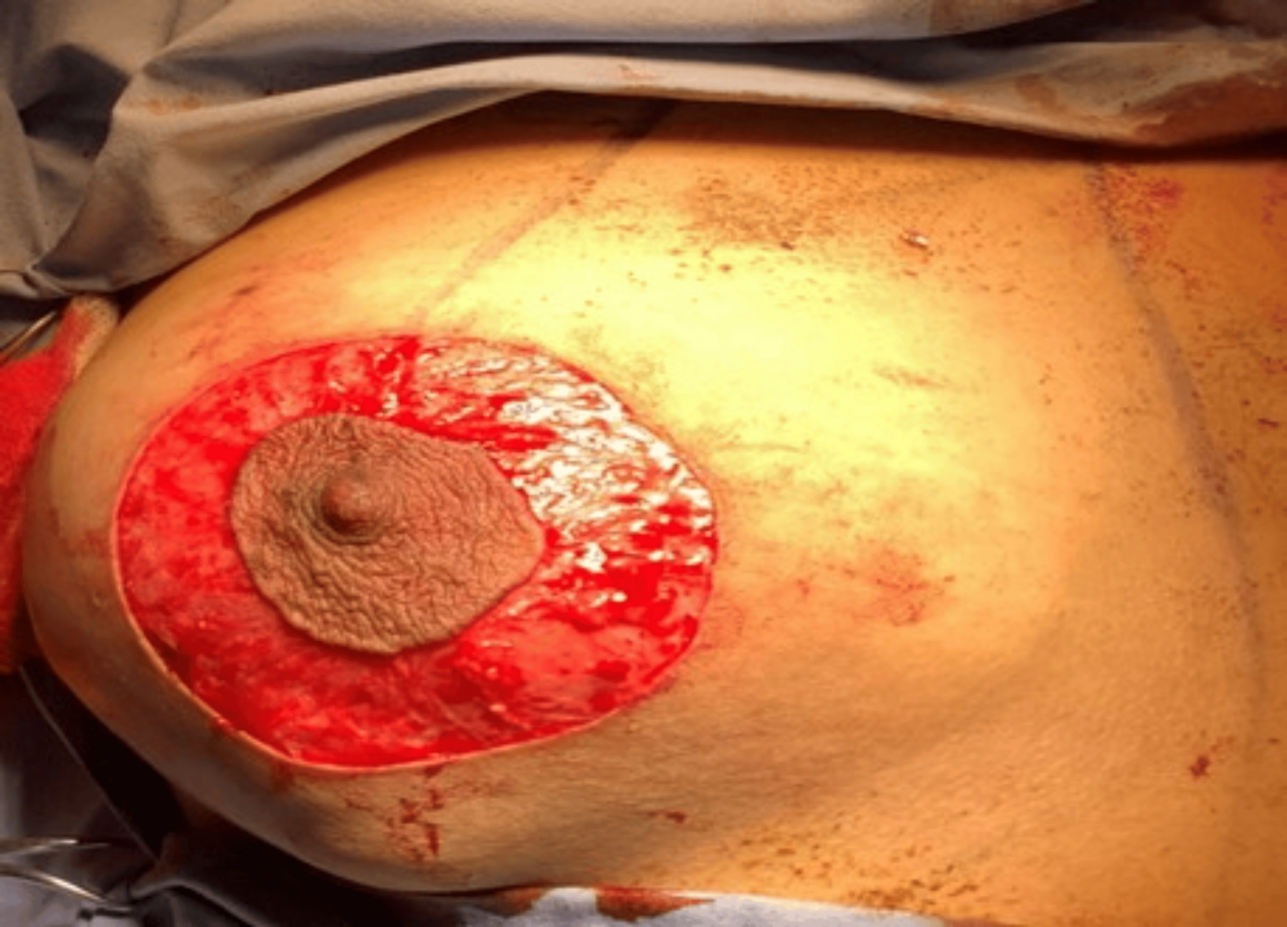
De-epithelization of the skin of the right breast

**Figure 5:**
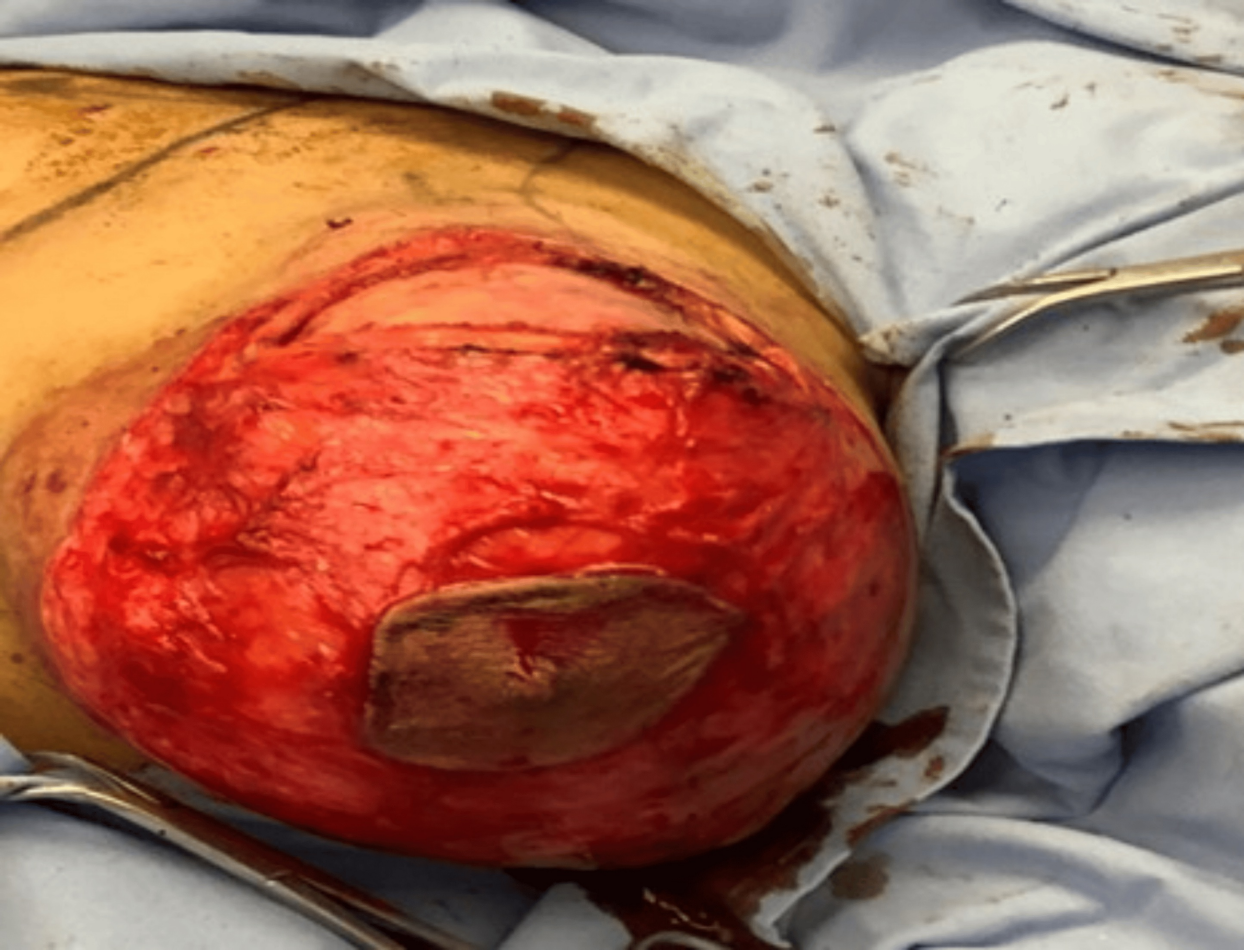
De-epithelization of the skin the left breast

After induction of general anesthesia and placing the patient in a supine position with both arms prepared and draped, a circumareolar skin incision was made to perform de-epithelialization of the skin followed by a direct incision over the largest mass (Figures 4, 5). 

**Figure 6:**
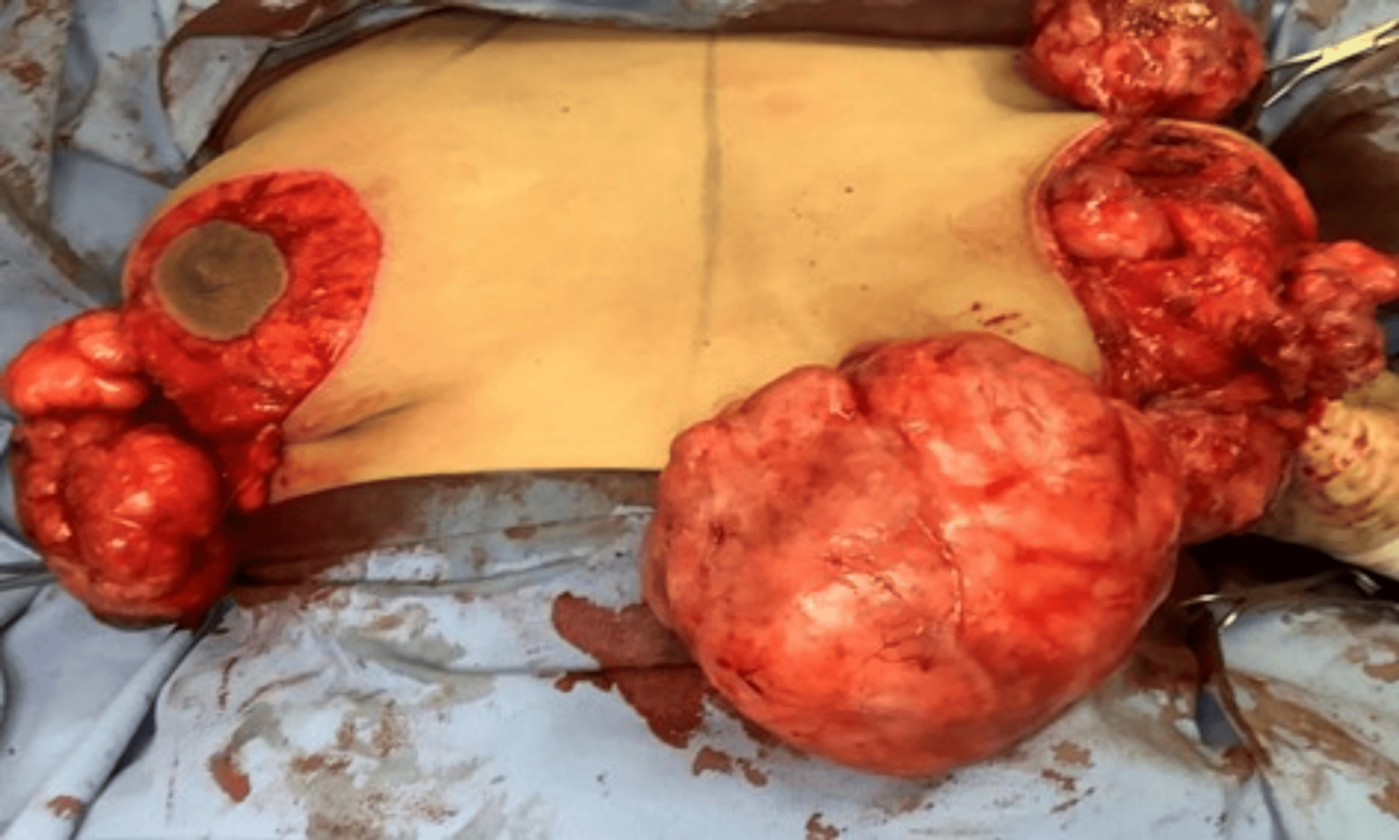
Intraoperative picture shows dissection of all fibroadenomas

**Figure 7:**
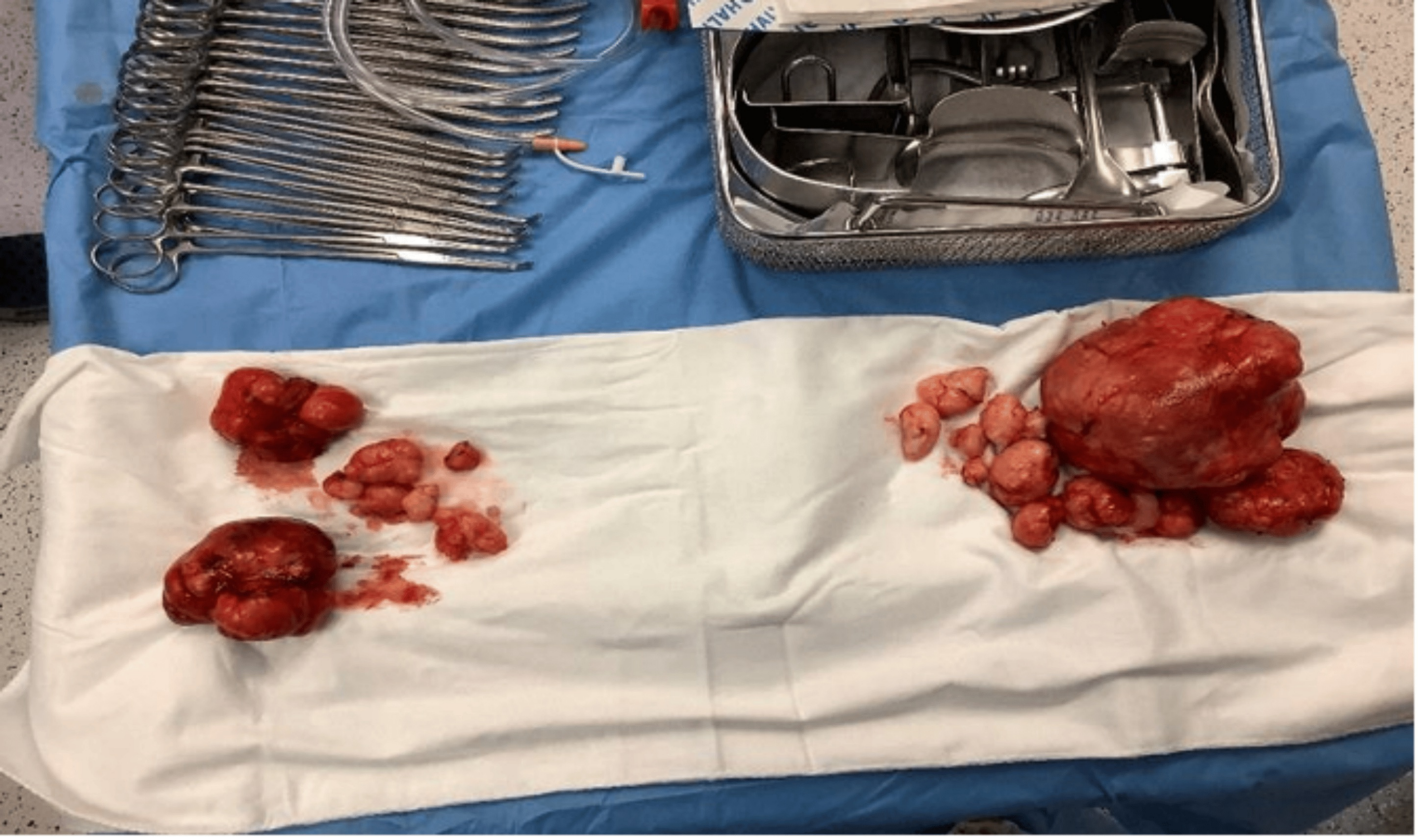
Specimens

Dissection down to the pectoralis fascia and the creation of a posterior plane was done to allow for access to all fibroadenomas, which were excised through a radial incision in the breast discoid tissue aiming to preserve the duct system (Figures 6, 7). 

**Figure 8:**
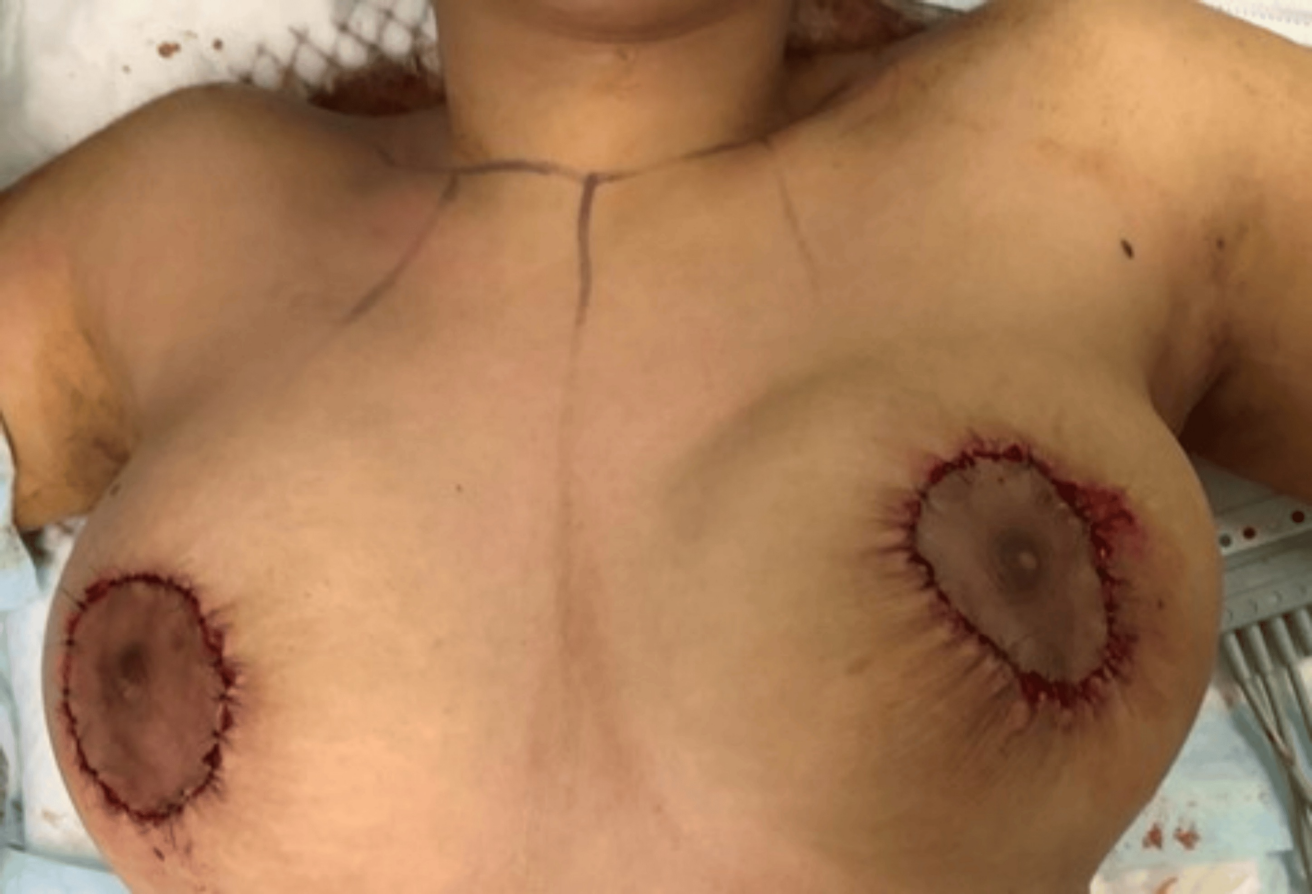
Closure of the round block incision

Drains were not inserted to preserve the breast tissue and let possible seroma reshape the breast (Figure 8). 

**Figure 9:**
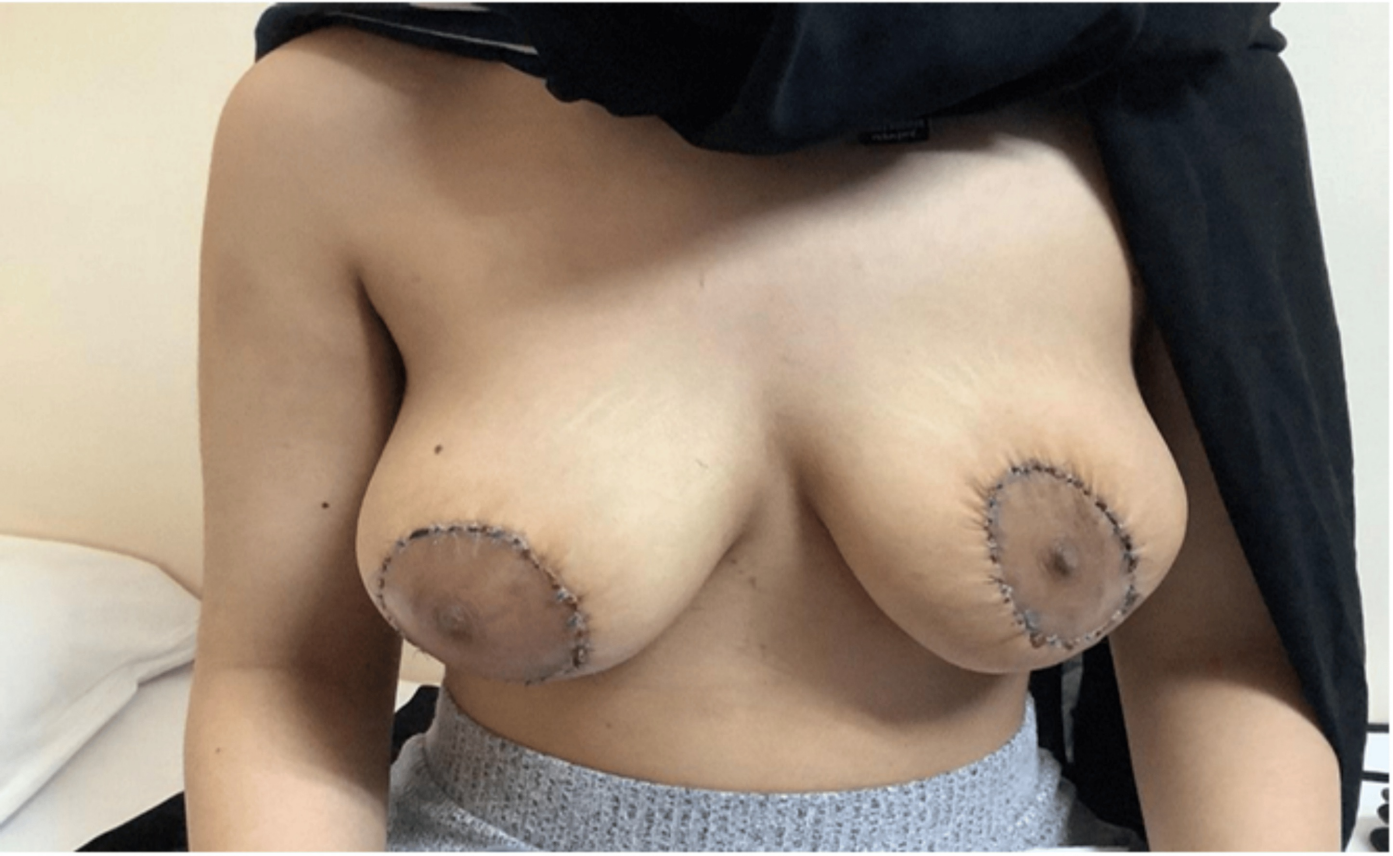
Post-operative picture after removal of stitches

Postoperative care was uneventful, and the scar was acceptable to the patient. Our patient was discharged from the hospital on day one, and we did not insert drains. The sutures were removed after two weeks, and photographs were taken. The vascularity and sensation of the nipple and areola complex, as well as the shape and position of the breasts, were satisfactory (Figure 9).

**Figure 10:**
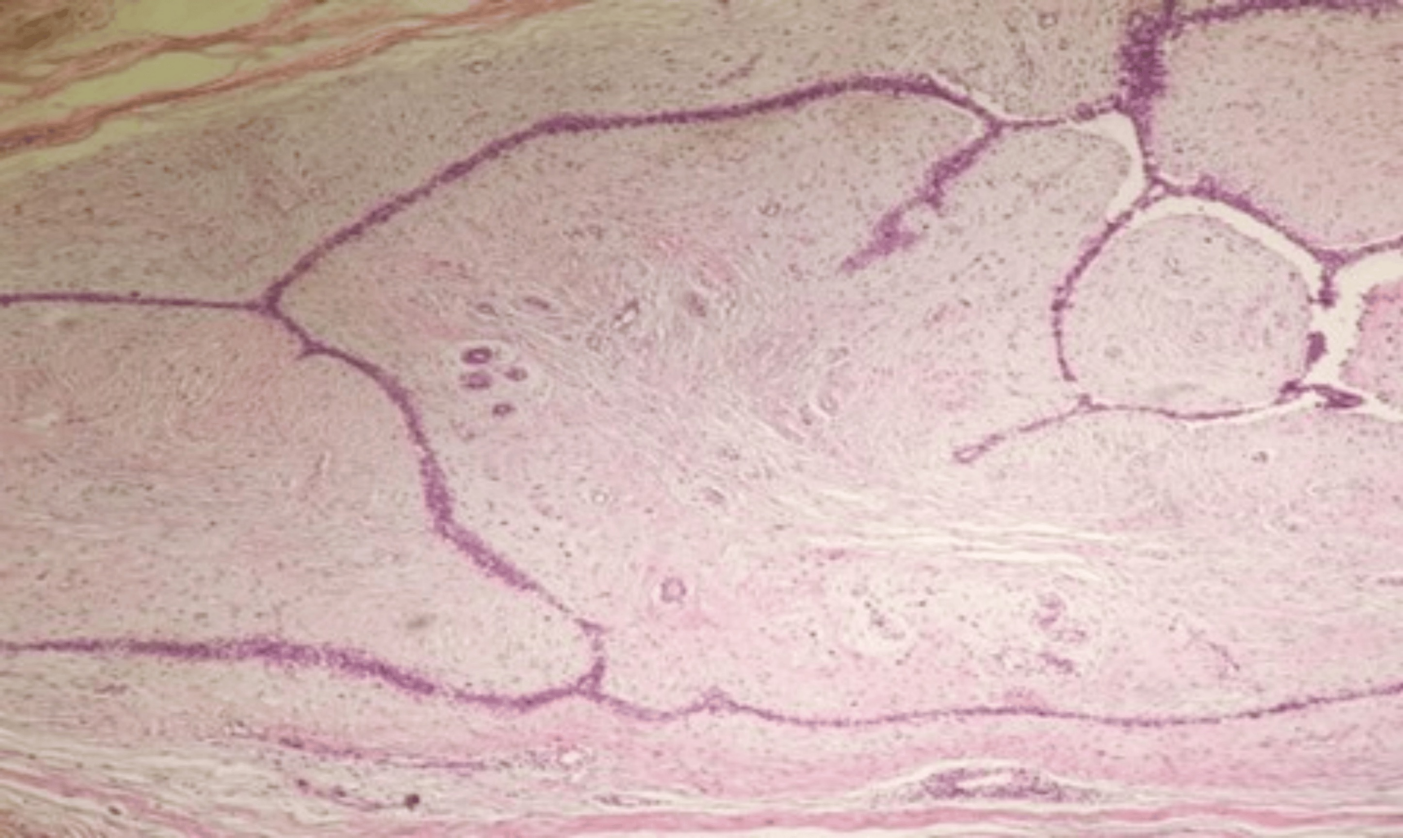
Histopathology image Haematoxylin and Eosin (H&E stain). The original magnification is 100X.

**Figure 11:**
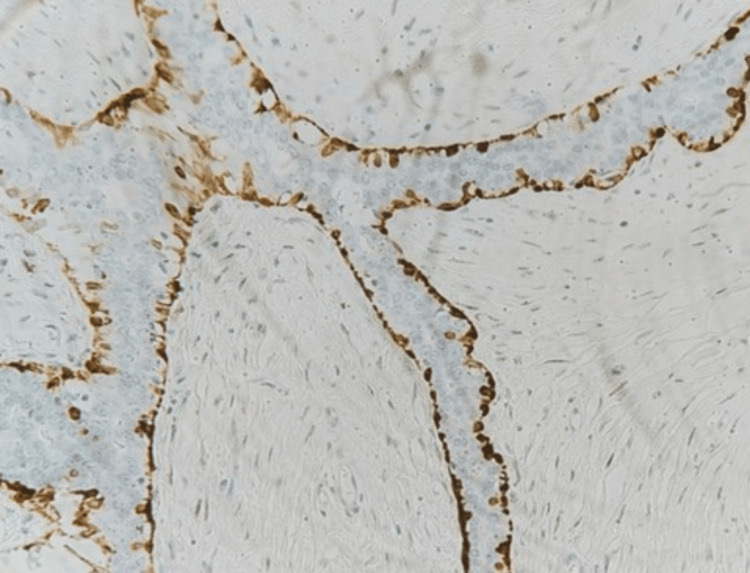
Calponin highlights preserved myoepithelial cells Original magnification is 400X.

**Figure 12:**
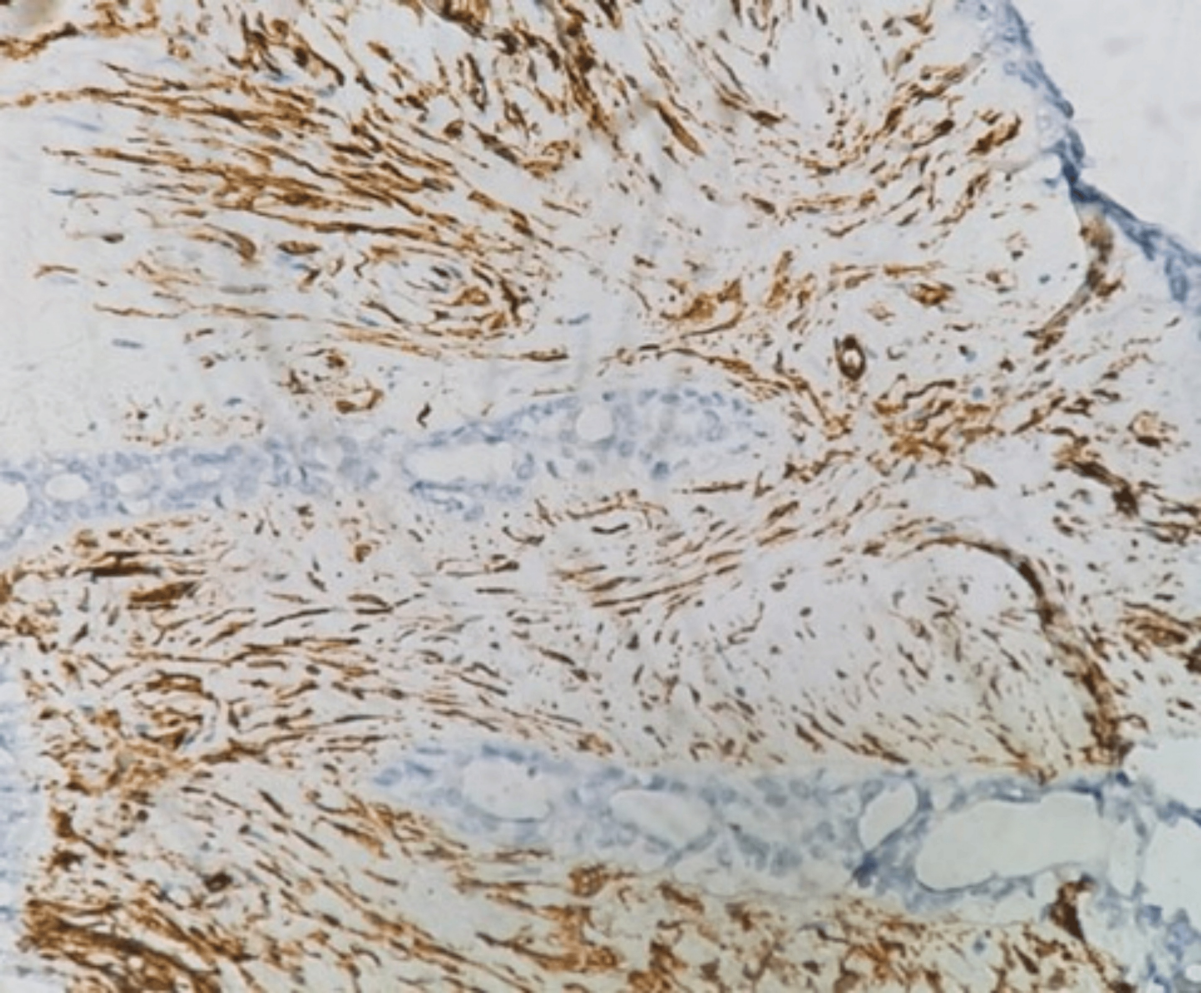
CD34 stains stromal cells Original magnification is 400X.

Histopathological examination and immunohistochemistry staining were done (Figures 10-12) to exclude other lesions, such as phyllode tumors or invasive carcinomas. 

Our patient said, "I am so happy with the results; especially, I was so desperate as I had been told by many plastic surgeons over two years that the only solution was the removal of both my breast and reconstruction could be made either in the same setting or after it by silicone insertion. I cannot afford such an operation, especially the reconstruction part; in addition, I would not be able to breastfeed later on, and I would not have normal breast sensations anymore. I am surprised by the results: no obvious scar, shape, or size of my breast after the left one was pendulous; now, they are nearly at the same level.

## Discussion

Multiple fibroadenomas are considered relatively uncommon even though fibroadenomas are the most common benign breast tumors in women between 15 and 35 years of age [1]. The etiology of multiple breast fibroadenomas has not yet been fully understood. Oral contraceptive pill usage is one possible cause. Other theories consider an imbalance of estrogen levels, breast receptors that are hypersensitive to estrogen, dietary factors, or inherited genetics. The increased sensitivity to estrogen may subsequently cause mammary gland hyperplasia and even carcinoma development. Therefore, patients with fibroadenomas may have a slightly increased possibility of developing breast cancer [3]. The literature regarding multiple bilateral breast fibroadenomas is scarce. Samala and Gedam reported 12 unilateral breast fibroadenomas [4]. Panda et al. detected 27 bilateral fibroadenomas in a 46-year-old patient [5].

Triple assessment is used as usual for any breast lump: clinical evaluation, imaging, and histological analysis. Fibroadenomas are usually well-circumscribed lesions that are distinct from the surrounding tissues. The diagnosis can be suspected on a clinical basis. Excision of fibroadenomas can be done under either local or general anesthesia. The optimal incisions are circum-areolar and through the inframammary crease to minimize visible scarring; however, the size and location of the mass may ultimately guide the incision location and length. Regardless, multiple breast lump excision is more difficult because the aim is breast conservation through the same scar [5]. The semicircular sub-mammary Gaillard-Thomas incision is usually used for multiple fibroadenomas and is made at the margin of the breast, where lumps can be removed without making multiple incisions. The Ribeiro and Rezai technique, which uses the inferior pedicle, allows excision in any part of the breast and remodeling [6]. In our case, we used bilateral round block mammoplasty to conserve the breast tissue as the patient has a desire for normal breastfeeding, which would not be possible after bilateral subcutaneous mastectomy and implant reconstruction. The round block technique is a useful oncoplastic procedure for the management of multicentric fibroadenomas excised at the same time [7].

This case report adds to the available data on multiple breast fibroadenoma management. It also demonstrates the usefulness of the round block incision in multiple breast lump removal because it gives a wide range of access to all breast quadrants and breast reduction in the same setting with local advancement flaps of superior and inferior breast tissue to achieve good cosmetic results.

## Conclusions

Bilateral multiple breast fibroadenomas are uncommon. The uniqueness of our case lies in the patient’s desire for normal breastfeeding, the pendulous left breast, and the huge anatomical size and site of the fibroadenomas. The round block incision gives wide access for the removal of multiple breast lumps with good cosmetic outcomes in the case of ptotic breasts with redundant skin due to the mass effect of the huge fibroadenomas and breast tissue preservation. Surgical excision is challenging and requires significant effort to conserve the breast and achieve better cosmetic results. To overcome these challenging surgical requirements, breast surgeons should not only be experts in basic general surgical techniques but also have some knowledge of relevant basic plastic surgery concepts.
